# Dental Radiographic/Digital Radiography Technology along with Biological Agents in Human Identification

**DOI:** 10.1155/2022/5265912

**Published:** 2022-01-18

**Authors:** Mohsen Yazdanian, Shahryar Karami, Elahe Tahmasebi, Mostafa Alam, Kamyar Abbasi, Mahdi Rahbar, Hamid Tebyaniyan, Reza Ranjbar, Alexander Seifalian, Alireza Yazdanian

**Affiliations:** ^1^Research Center for Prevention of Oral and Dental Diseases, Baqiyatallah University of Medical Sciences, Tehran, Iran; ^2^Department of Orthodontics, School of Dentistry, Tehran Medical Sciences, Islamic Azad University, Tehran, Iran; ^3^Department of Oral and Maxillofacial Surgery, School of Dentistry, Shahid Beheshti University of Medical Sciences, Tehran, Iran; ^4^Department of Prosthodontics, School of Dentistry, Shahid Beheshti University of Medical Sciences, Tehran, Iran; ^5^Department of Restorative Dentistry, School of Dentistry, Ardabil University of Medical Sciences, Ardabil, Iran; ^6^Science and Research Branch, Islamic Azad University, Tehran, Iran; ^7^Nanotechnology and Regenerative Medicine Commercialization Centre (Ltd), The London Bioscience Innovation Centre, London, UK; ^8^Department of Veterinary, Science and Research Branch, Islamic Azad University, Tehran, Iran

## Abstract

The heavy casualties associated with mass disasters necessitate substantial resources to be managed. The unexpectedly violent nature of such occurrences usually remains a problematic amount of victims that urgently require to be identified by a reliable and economical method. Conventional identification methods are inefficient in many cases such as plane crashes and fire accidents that have damaged the macrobiometric features such as fingerprints or faces. An appropriate recognition method for such cases should use features more resistant to destruction. Forensic dentistry provides the most appropriate available method for the successful identification of victims using careful techniques and precise data interpretation. Since bones and teeth are the most persistent parts of the demolished bodies in sudden mass disasters, scanning and radiographs are unrepeatable parts of forensic dentistry. Forensic dentistry as a scientific method of human remain identification has been considerably referred to be efficient in disasters. Forensic dentistry can be used for either “sex and age estimation,” “Medical biotechnology techniques,” or “identification with dental records,” etc. The present review is aimed at discussing the development and implementation of forensic dentistry methods for human identification. For this object, the literature from the last decade has been searched for the innovations in forensic dentistry for human identification based on the PubMed database.

## 1. Introduction

In the past decade, an alarming rise in criminal and casualty incidences has been observed [1]. The contemporary considerable prevalence of violent and criminal actions has necessitated applying modern methods for criminal investigations [2]. Additionally, the present incidence of casualties associated with mass disasters (MD), such as travel and transport accidents, terrorism, and unusual climatic conditions, needs novel efficient methods for MD victim identification [3]. The legal discipline of using medical facts for victim identifications is called “forensic medicine” [4]. Forensic has a Latin root in the term of *forense* or *forensis* which means a forum, public, or marketplace where the legal issues are discussed [2]. Forensic dentistry or forensic odontology is defined as the knowledge of dentistry as related to the law and is one of three primary identifiers recognized by Interpol for victim identification in multicasualty incidents or MDs [2, 5]. The scanning techniques including different types of digital radiographs and photographs have an approval role in forensic dentistry [6]. The diversity of dental patterns among human individuals has facilitated the accurate identification of them [7]. It has been said that one of the most common AM evidences applied for human identification is their dental radiographs which have a crucial role in forensic sciences [8]. Moreover, some macrobiometric features are recognizable when the entire skeleton is available which helps for example identify the victim gender with 100% accuracy. However, in most MD cases, the victim bodies are more damaged than be visually identifiable. The modern identification processes are very helpful in such cases. These methods use microscopy and molecular examinations of the remained sources which often include the skull and teeth [9]. Using forensic dentistry as a proven reliable scientific method in legal identification cases dates back to the 1950s and 1960s in the developed countries of Europe and the United States [10]. Since then, using forensic dentistry has been extensively helpful for human identification especially in MDs, and progressing the new methods has made its application increasingly more applicable [11]. Not only do dental identification mainly benefits from detection of damages inflicted to the jaws, oral tissues, and teeth, it can help to suspect elimination or potential identity [12]. Forensic dentists are required to process, review, and evaluate the collected evidence from dental remains in the form of scientific and objective data and present them to legal authorities [13]. Forensic dentistry has significantly changed in the past decades from being occasionally used to playing a key role in identification procedures [14]. Since it is simple, user-friendly, and not expensive in comparison with other methods, forensic odontological comparison is considered one of the three principal identifiers designated by INTERPOL for use in identifying the victims of a multicasualty incident. Its positive outcome is considered sufficient to permit personal identification without further support from other methods [15]. The present review study is aimed at having a systematic survey on the role of forensic scanning technologies in the identification of individuals contributed to a crime or disaster. Recent human identification researches in the field of dental records are shown in Table 1.

## 2. Dental Parameters Used in Sex and Age Estimation

Age, sex, and race are the very basic and fundamental characteristics generally used for defining and identifying every human individual [16]. The age of children up to puberty can be estimated from the developmental stages of their teeth in dental radiographs and scanning since they are minimally influenced by malnutrition and hormonal and pathological disorders [17, 18]. Among different techniques used in forensic science, scanning ways are the hallmark [19]. Parameters that show “sexual dimorphism” are very helpful for determining the sexuality of the victims. Sexual dimorphism is the considerable differences that a parameter shows in size, stature, and/or appearance depending on sexuality. The second important variable required to be determined within the biological profile of a victim is their age at the time of being missed or dead. The recovered skeletal remains represent classic features applied for this purpose [20]. Except for bone morphology, age can be estimated in a range of decades using dental findings too [21]. In the following, dental parameters that help to identify the sex, age, and race of an unknown person are listed.

### 2.1. Radiographic Estimation

For this aim, three methods are used. First is the age determination using a developmental process to wit clinically visualization of formation, eruption, and calcification of teeth [18, 22]. The second method is radiographically using intraoral periapical radiographs, bitewing radiographs, and OPG. The radiographic images must include developing teeth of interest to be evaluated based on the selected development standards [23]. The third and last method is the radiographic staging technique of the mandibular third molar tooth development using the DI combined with the skeletal maturity using radiographs from hand wrist and CVMI [22, 24]. Since accurate age determination becomes increasingly difficult after developing the third molar, the aging procedure and regressive conversions of teeth are the only helpful assessing methods at adult age [22]. A variety of structures can help to reach radiographic estimation. From different types of techniques for achieving this goal, the evaluation of third molar, maxillary and frontal sinus and cervical vertebral maturation are the most practical one which can raise the accuracy of estimation.

#### 2.1.1. Development of the Third Molar

Third molars and their radiographic images are used extensively for age assessment in the range of 9-23 years because of their long periods of the crown and root developments [25]. Third molar mineralization is frequently completed before 21; however, it has been observed to be completed before 18 in some populations [26]. Assessing the maturity of the third molar is a noninvasive method recognized as the best age estimation way because its radiographic images can be easily obtained. This is while no radiographic images from many other skeletal aging indicators are available [27]. Third molar development can be also explored by CT scans and X-ray free imaging MRI methods. These methods are noninvasive and practical and present 3D images of the third molar. Also, geometric distortion does not make any magnification errors in them. MRI is mostly used in countries where radiation is not recommended or allowed [28].

#### 2.1.2. Maxillary Sinus

**Figure 1:**
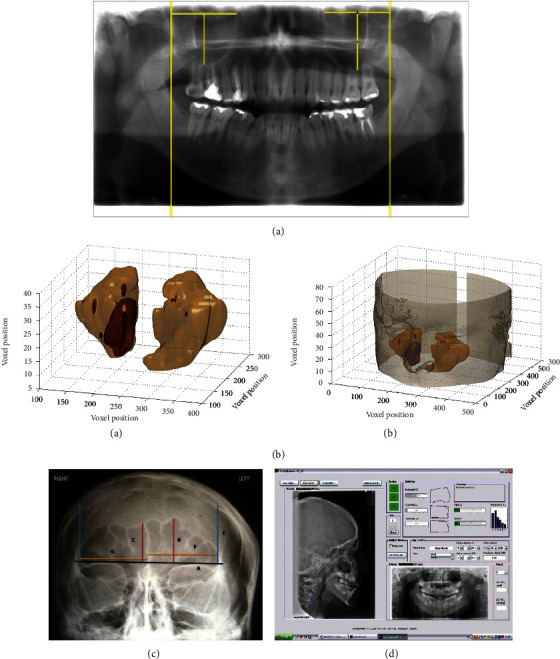
A panoramic radiograph that reveals the measurement of maxillary sinuses' width and height (a) (reprinted with permission, *Acta Stomatologica Croatica*) [32]. Reconstruction of 3D shaded surface of the maxillary sinuses (beige surface), highlighting maxillary sinus involvement (brown surface): (A) maxillary sinuses, (B) maxillary sinuses inside the reconstructed head (b) (reprinted with permission, *PLOS ONE*) [31]. Diagram of Caldwell with the demarcation of borders of the frontal sinus and identification of the measurements: (A) baseline, (B) maximum left height, (C) maximum right height, (D) lateral most point of the perimeter on the right side, (E) lateral most point of the perimeter on the left side, (F) maximum left width, and (G) maximum right width (c) (reprinted with permission, *Wolters Kluwer-Medknow*) [36]. The GUI of the computer application Cephalometar HF V1 (d) (reprinted with permission, *Academy of Medical Sciences of Bosnia and Herzegovina*) [40].

MS is the first among the paranasal sinuses (frontal, ethmoid, maxillary, and sphenoid sinuses) that develop (at 10 weeks of intrauterine life) [29]. The dimensions of the maxillary sinuses are considered reliable indicators for age determination since they are interestingly shown to remain intact in accidents and casualties such as explosions, warfare, and aircraft crashes, where the skull and other bones are mostly disfigured terribly [30]. MS volume can be under the influence of several environmental and physiological factors such as breathing patterns, dental problems, the anatomy of the body, gender, age, ethnicity, and climate [31]. In cases of incomplete skeletons, the ratio of maxillary sinus width and height to other bones can be used for sex identification.  Figure 1(a) shows the method of measuring the width and height of the maxillary sinuses [32]. Traditional X-ray technique has been mostly substituted by CT and MRI for imaging the paranasal sinuses [30]. CT is a popular method for MS imaging [33] and brings out a 3D shaded image of the MS surface (Figure 1(b)) that illustrates MS involved inside the reconstructed head (Figure 1(b)) [31]. For viewing the MS, radiographs are mostly taken from the occipitomental view (Waters' projection) of the skull [34].

#### 2.1.3. Frontal Sinus

The frontal sinus [35] is a highly unique tissue that rarely changes throughout life that facilitates identity assessment using linear and area measurements in all genders [36]. The frontal sinus also barely endures aplasia providing a reliable assessment through comparing antemortem and postmortem radiographs of FS [37]. Different FS measurements are shown in the Caldwell view (Figure 1(c)) [36]. Posteroanterior (PA) radiograph provides the best view of the frontal sinus by the Caldwell technique. Lateral cephalograms, CT, and CBCT are other techniques occasionally used for studying the frontal sinus [34].

#### 2.1.4. Cervical Vertebrae

Another indicator for assessing the bone development extent is the maturation degree of CVM [38]. The modification in the size and shape of cervical vertebrae (mostly second, third, and fourth vertebrae of the neck) in both genders ideally represents the pubertal growth period of craniofacial bones during adolescence and young adulthood [39]. The morphological analysis of these vertebrae (C2, C3, and C4) is used for assessing for example the lower border concavity, and the CV body shape changes during skeletal maturation [40]. Cephalometar HF V1 is another reliable method for assessing the CVM stages. The GUI of the application is shown in Figure 1(d) [40].

### 2.2. Mandible

The mandible or lower jaw is the largest and toughest bone in the face that forms the lower part of the skull. The mandible is a horizontally curved body having two rami and a convex ascending from the posterior to the anterior of the face [41]. Mandibular morphological parameters such as the gonial and antegonial angles of mandible, mental and mandibular foramen, as well as the mandibular canal, change during life and between genders [42]. According to Albalawi et al., the lines from the left and right gonion to menton (Gn-M0) form an angle that provides helpful anthropological data for dental and medicolegal practices [41]. Gn-M0 angle and minimum ramus breadth and height show statistically significant dimorphism between the two genders making them reliable indicators for sex demarcation [43].

#### 2.2.1. Mental Foramen

MF appears in different shapes (round, oblong, slit-like, or irregular) in radiographs. It is a partially or completely corticated radiolucent area [44] that shows significant changes in height according to age [42]. OPGs facilitate surveying the complete mandible leading in a more accurately determining the vertical and horizontal measurements of the mental foramen [45]. The CBCT technology is increasingly used for the 3D locating of MF because of its magnification-free high-resolution imaging potential and precision [46].

### 2.3. Cementum

**Figure 2:**
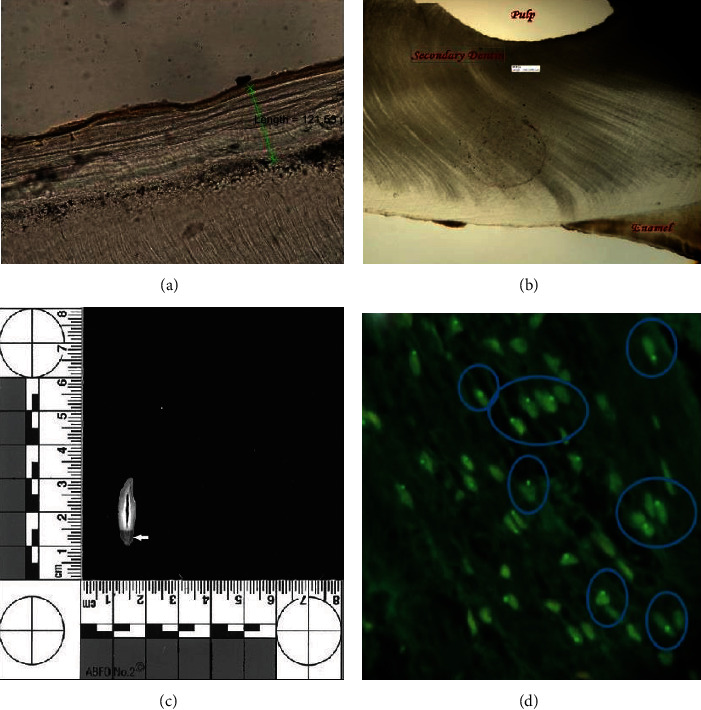
A ground tooth section represents the cementum width from the dentinocemental junction to the cementum surface (a). A ground tooth section representing the secondary dentin thickness at the coronal third of root (b) (reprinted with permission, JCDR) [51]. Scanned image of a tooth section with American Board of Forensic Odontology no. 2 scales. The translucent dentin shown by the arrow has emerged as a dark area (c) (reprinted with permission, *Wolters Kluwer-Medknow*) [53]. Acridine orange has positively dyed cells from feminine samples because of the presence of Barr bodies (d) (reprinted with permission, *Wolters Kluwer-Medknow*) [57].

The calcified tissue around the teeth which contains the periodontal ligament fibers is an acellular part of the dentine called cementum. Cementum forms the attachment site of teeth to the part of the jaw that holds them which is known as the alveolar bone [47]. The continual construction process of cementum and its conserved model during life has made cement-chronology possible. Cementochronology is a potent method for directly evaluating the chronological age and determining the season at death [48]. The apical areas of cementum are thicker than parts at the CEJ, and its shape and texture are stable within an individual's life [49]. The underlying mechanism is the racemization reaction of aspartic acid in a constant manner [50] that produces a connective tissue in the form of incremental layers surrounding tooth roots and creates an appearance of concentric lines [51]. These circular lines are called salter lines, and each pair of them represents one year of life (Figure 2(a)). The number of salter lines provides a biological record that represents the estimated age of the victim [51].

### 2.4. Dentine

The secondary dentin starts forming after completing the tooth root and primary dentine. Since secondary dentin formation is a continuous process throughout life, its amount is applicable for age estimation. Some physical or chemical insults or dental caries can affect the regularity of the secondary dentin (Figure 2(b)) [51]. One of the applicable related biomarkers is the quantity of root dentine translucency that is determined using vernier calipers or digital aids [52]. Environmental factors and pathological processes minimally affect this parameter, and it can be macroscopically examined in both thorough teeth and sectioned teeth. Root dentine translucency appears symmetrically distributed on both sides of the jaws [52, 53]. An example of a scanned image of a tooth section is shown in Figure 2(c) [53].

### 2.5. Dental Pulp Chamber

The innermost soft core of pulp is protected by cementum, dentin, and enamel which is the outermost covering of the tooth crown. These tissues have a hard structure that is resistant to decomposition [54].

#### 2.5.1. Radiographic Aspect

A parameter in correlation with the chronological age of victims is the size of their pulp chamber that can be obtained from the radiographic examination of their teeth [54]. Since the volume of the pulp chamber changes during life [55], the pulp chamber volume is measured using the pulp/tooth area ratio method in both panoramic and periapical radiographs. Also, the PV/TV ratio and its relation with age are now extensively applied in clinical dental practice using 3D images [55]. According to Ravindra et al., the apical pulp area reduces as individuals get older in an increasing manner through age. The changes in the apical area are more obvious than in the middle area or the pulpal floor probably because of the age-related modifications of the cementum and dentine. In this regard, the related literature reports that despite the apical pulp area, the mean size of the middle and coronal pulp does not change [56].

#### 2.5.2. Histologic Aspect

The sex of a subject is histologically determined through exploring the presence of a sex chromatin body (Barr body) consisting of a condensed, inactive X chromosome mainly in the somatic cell nucleus. The Barr body can be observed in various cells using most of the nuclear stains and is regarded as representative of genetic femaleness (Figure 2(d)). The commonly used staining methods of Barr body include H/E, thionine, Papanicolaou, Feulgen, cresyl violet, Giemsa, aceto-orcein, and under fluorescence such as AO. Barr body analysis for sex determination is considered the most reliable method when the tooth is the only evidence that remained at the crime scene [57–60].

#### 2.5.3. Advanced Techniques

In addition to the above-mentioned examinations, the dental pulp tissue is also used for PCR analysis and AMEL identification. Both of these advanced techniques help determine the sex of victims. Dental pulp-containing fibroblasts are an excellent source of DNA [57]. From the authors' standpoints, as digital scans especially OPGs are more reachable and economical way than histologic analysis, they may be a more practical method in sex and age estimation. The above-mentioned parts illustrate the importance of scanning methods and even a single tooth in forensic science. Not only have the radiographs an important role in sex and age estimation, but also the presence of a single tooth can raise the accuracy of estimation dramatically.

## 3. Dental Records

The extreme hardness of teeth preserves them intact in a dead body for a long time [61]. Then, the AM dental data and material such as dental records, X-rays, CT scans, dental model, and full-face photographs can be obtained from the missing person's dental practitioner by the local police and get interpreted by a forensic odontologist [62]. Therefore, every dentist is required to accurately record all dental data and maintain them for any probable legal circumstances [63]. The unicity of dentition in every particular individual has made it a resistant analog to fingerprints [64] which is greatly useful in cases of highly demolished bodies for example by fire [65]. The reason is the hard tissue of teeth and dental restorations that is extremely resistant to destruction even in cases of charred and decomposed bodies [65]. Among the different techniques used in forensic dentistry, scanning ways are the most practical ones [19]. To use dentition records for identification, an official office document referred to as the patient's chart or dental profiles is developed that contains all patient dental treatments [66]. In the cases of mass casualties, a forensic odontologist is generally responsible for comparing the antemortem (AM) and postmortem (PM) data in the patient's chart and extracting the matches which support identification [15]. AM data are usually obtained from the private clinics where the victim has records of dental treatments such as radiography, prescription, dental casts, and photographs while the PM data is obtained during cadaveric examinations [67]. The most common AM evidence applied for human identification is their dental radiographs [8]. Recently, forensic dental evidence has helped to primarily solve many incident cases of rapes, trafficking, terrorist attacks, homicides, and natural disasters [1, 68].

### 3.1. Digital Radiography

**Figure 3:**
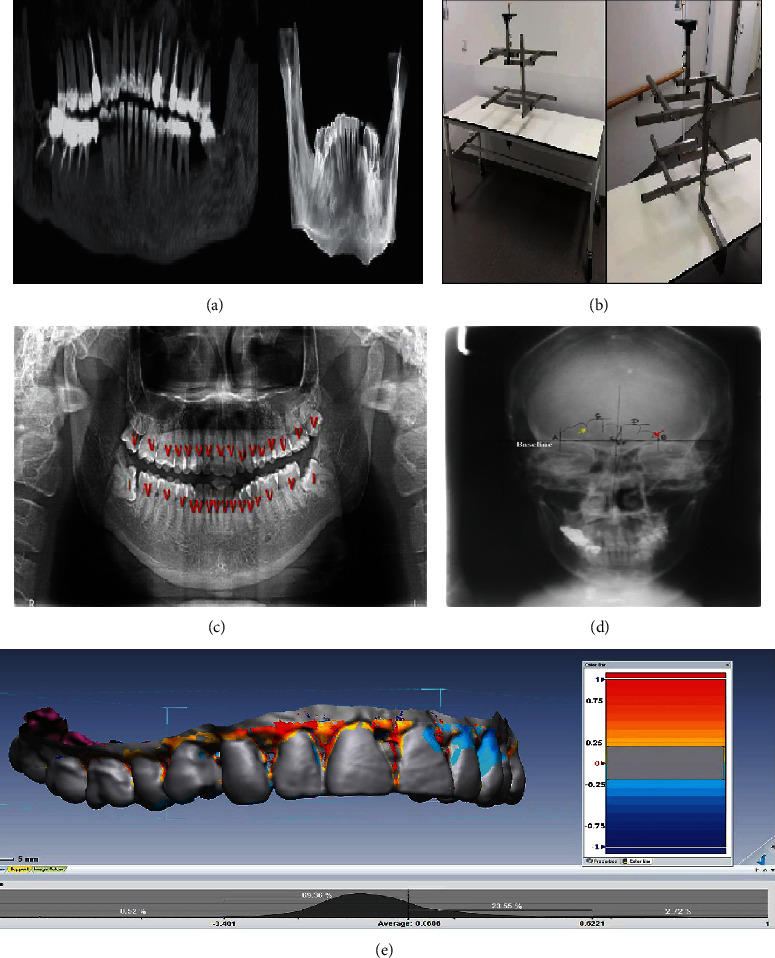
Dental reconstructions for identification applications are illustrated (left). A panoramic reconstruction with maximum intensity projection is represented including root canal fillings, metallic crowns, bridges, and missing teeth [158]. Volume rendering technique reconstruction of a mandible, with colored dental structures corresponding to metal crowns (a) (reprinted with permission, *BIR*) [69]. This fixation system has been created for PM DPR merging. Both tall and short bodies can be fixed reliably by adjusting the upright and the holding arms. Using chin rest is recommended for perfectly positioning the head (b) (reprinted with permission, *Springer Nature*) [75]. Panoramic radiograph with suitable annotations (commonly observed dental pattern) (c) (reprinted with permission, *Wolters Kluwer-Medknow*) [73]. This traced PA skull radiograph represents the borders of sinus and metric variables. The red and yellow arrows point to the scalloped arcade and the partial sinus line, respectively. Point A to B indicates the maximum width of the frontal sinus. The baseline to point C indicates the maximum height of the frontal sinus (d) (reprinted with permission, *I.O.F.O.S.*) [78]. 3D superimposition: the PM surface is superimposed on the AM surface (e) (reprinted with permission, *Taylor & Francis Group*) [15].

The diversity of human dentition comes from the number of teeth [32] in 6 different types (incisor, lateral incisor, canine, premolar, and molar) and the unique dental treatment which is not the same between every two persons [7].  Figure 3(a) illustrates the comparison points in the dental radiographs for identification which include the number and arrangement of teeth, dental anatomy, caries, coronal or hidden restorations, periodontal bone loss, bone pathology, trabecular and crestal bone topography, nutrient canals, anatomical landmarks of the teeth and jaws, and the size and morphology of both maxillary/frontal sinuses and nasal aperture [69]. Teeth that are missed, rotated, spaced, extra, and/or impacted can affect the dentition number and arrangement. Bases under fillings, pins, root canal fillings, posts, and implants are the hidden features that are only seen by radiography [8].

#### 3.1.1. Orthopantomography (Panoramic Radiography)

Orthopantomography or panoramic radiographs are the panoramic X-ray scanning of the upper and lower jaws that provide a broad radiologic graph from the human dentition. Because of the low radiation dose and short time required for imaging, dental panoramic radiography [70] has become a broadly applied standard method in dentistry as complementary information and initial examinations for oncological treatments [71–73]. Accordingly, orthopantomography is now practically used for victim identification in several MDs including war casualties [74]. During recording PM data, the corpse fixation system allows acquiring a reliable panoramic graph (Figure 3(b)) [75]. In Figure 3(c), the most commonly observed dental pattern is illustrated [73]. Even though OPGs are one of the routine methods in human identification, since they have a lot of effective information, the only limitation in using the panoramic technique is its sensitivity to misalignments of the body which may lead to image distortion [76].

#### 3.1.2. Lateral Cephalometry

A lateral cephalogram is an X-ray of the craniofacial area from the lateral angle that displays a variety of anatomical points and distinctions of architectural and morphological structure and intra-cranial details, simultaneously. Therefore, it has an exemplary application in skull analysis and evaluation [77]. According to Sassouni, some niceties such as bigonial width, cranial height from the mastoid to vertex, bimaxillary breadth, height from bi-gonial width to temporal crest, maximum cranial breadth, frontal sinus breadth, incisor height, and facial are referred to be evaluated in lateral skull radiographs and compared in the ante and postmortem records [74]. As it is said in the previous part, like OPG, lateral cephalometry is sensitive to misplacement of the head in the head position part of a machine. Also, magnification of AM and PM images may result in distortion and it should not be neglected in human identification.

#### 3.1.3. Frontal Radiography (Posteroanterior or PA Cephalometric Analysis)

Frontal sinuses or PA cephalometric analysis are other individual traits known for their high variations among people that make them suitable evidence for forensic purposes [74]. Pattern matching in PA cephalometric analysis is performed using both Caldwell-oriented film projections and occipitomental or Water's view. The frontal sinus is better displayed in Caldwell's view while Water's view provides a slightly foreshortened image. According to Nikam et al., the variables of frontal sinus can be measured for forensic identification by drawing a certain tangent to the baseline and dividing the sinus area into four parts. The general sinus variables include the number of complete sinus cavities, number of partial sinus lines, maximum overall height above baseline, maximum overall width, and number of complete sinus cavities left of the septum, as well as some partial sinus lines in the main cavity, number of scalloped arcades on the main cavity, the maximum height of quadrant above baseline, maximum height of the main cavity above baseline, maximum width of the main cavity from the tangent line, and maximum width of the main cavity on both left and right sides of the sinus (Figure 3(d)) [78].

#### 3.1.4. Cone Beam Computed Tomography (CT)

Another frequently used method in forensic investigations for providing AM data is computed tomography known as CT [79]. MSCT or cone beam images can produce a panoramic or 3D image of teeth, peripheral soft tissues, nerve pathways, and connected bones in a single scan by some posttreatment processing [69]. The AM 3D scan data can be compared with the PM data from CT or CBCT scans or a 3D scanner (Figure 3(e)) [15]. Some newly developed software such as Dentascan (GE Health care, UK) has helped more precise identification with fewer artifacts by reformatting the panoramic images [74]. However, several factors such as cone beam or dental restorations can produce different types of CT artifacts which can impede comparison between AM and PM radiographs [80]. Dental reconstructions cause artifacts because of their increased radio-opacity [69].

### 3.2. Forensic Digital Photography

Especial devices such as digital cameras are developed for recording PM dental information such as oral photographs and mouth gags [6]. Generally, forensic photography using cameras needs to follow certain rules, including securing the crime scene for providing proper evidence, evaluating the conditions (e.g., light, weather, and camera settings), shooting the entire scene using both wide-angle and close up shots for showing the relationship among pieces of evidence, recording the location, injuries, and condition of victims, using the right angles and eliminating probable distance distortions, and locating evidence markers using the first shot of entire crime scene. Photographers need to use alternate light sources such as lasers, blue/green lights, and colored filters to detect fingerprints, bite marks, and footprints [81].

#### 3.2.1. Facial and Intraoral Photographs

In forensic cases of human abuse and bite mark analysis, facial and intraoral photography is the most common and easiest diagnostic method of obtaining and maintaining evidence [82]. In cases that the face of the deceased is recognizable, the oral photographs can be applied for direct identification, while in cases of completely disfigured faces, the intraoral photographs are more applicable, because the intraoral photographs can display certain useful data about the hard tissue such as fluorosis, abrasion, tooth attrition, enamel decalcification, enamel cracks and fractures, and lower canine anatomy [83]. Also, smile photographs are significant evidence of the victim's teeth through life [84].

### 3.3. Dental Appliance and Restoration

Dental appliances and accessories such as full or partial dentures, decorative accessories or orthodontic appliances, bleaching trays, occlusal splints, and mouth guards are also used to identify the dentition or mouth of a specific victim or suspect [15].

#### 3.3.1. Denture Marking

**Figure 4:**
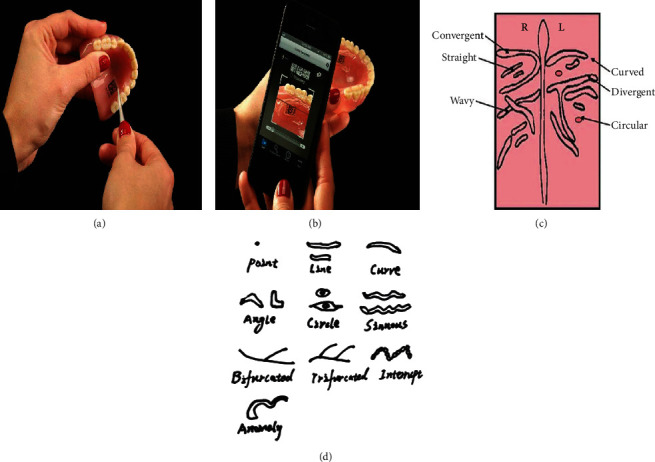
Martin dos Santos classification (a). Thomas classification (b) (reprinted with permission, *Wolters Kluwer-Medknow*) [92]. The DPid code is implanted in a denture that can be scanned using a smartphone (c, d) (reprinted with permission, *SciELO*) [87].

The denture marking of prosthetic and orthodontics appliances is now considered very important evidence for forensic identification in different medicolegal issues [85]. Denture marking is also applicable in nonforensic cases such as identifying a lost denture wearer suffering from amnesia or senility, loss of memory, and other psychiatric cases. Also, a denture with marking can be conveniently returned to the owner if it is lost and found. Regarding forensic issues, denture marking has an important role in identifying the unknown cases of homicide and suicide, as well as victims of fire, explosion, floods, earthquake, plane crash, or war [86]. Therefore, denture labeling provides a rapid and reliable method other than fingerprinting for identifying unknown individuals in the laboratory. For this aim, the denture marking is preferred to contain the name of the owner with or without other identifiers such as social security number, driver's license number, and city code [86]. Also, a coding method is invented by Queiroz et al. referred to as the DPid system which randomly generates individual 2D data matrix codes along with a 5-digit alpha/numeric token which is individually unique. Forensic investigators can scan this code by either a smartphone or a tablet equipped with a 2D Code Reader App. There is also a DPid website that authorities can log into and enter the patient's unique code and identify the denture device owner. The DPid system has been proved to be an efficient tool for solving forensic cases involving dental prosthesis (Figures 4(c) and 4(d)) [87].

#### 3.3.2. Dental Restoration

The country or region where the dental restoration has been performed can be recognized from the quality and type of treatment and materials. For example, in Central and South America, the anterior teeth are prevalently restored using silver or gold color metal crowns. While in Eastern Europe, these teeth are frequently restored using full cast metal crowns with acrylic facings [14]. Also, according to many dental anthropological studies, the morphological characteristics of teeth can be used for determining the race of the victim. For example, the Carabelli cusp, having few dental cusps, and simplified fissure patterns can be indicators of a Caucasoid race, while Asians are characterized by shoveled incisors and complex fissure patterns with the normal count of dental cusps [62].

### 3.4. Dental Cast

The dental cast provides a 3D model of both maxillary and mandibular arches that facilitate evaluating the malocclusions, morphology, and anatomy of the victim's teeth. These models are certainly proper for assessing the abrasions, attrition, and fractures in the enamel [83]. Also, using dental casts, certain odontometric parameters such as mandibular and maxillary canine indices, the size of mandibular canine, maxillary canine, and maxillary first molar, as well as the cumulative size of all teeth are used for determining the gender of the deceased [88].

#### 3.4.1. Palatoscopy (Palatal Rugoscopy)

Palatoscopy or palatal rugoscopy is the knowledge of palatal rugae that can be much helpful in cases of fingerprint unavailability such as decomposed and burned bodies [89, 90]. Different tissues such as the lips, cheek, tongue, and the buccal pad of fat, teeth, and jawbones preserve the internally located palatal rugae from trauma and high temperatures [91]. As shown in Figures 4(a) and 4(b) respectively, two main methods of classification for identification purposes have been suggested for the rugae patterns: Martin dos Santos classification (1983) and Thomas classification (1983) [92]. On the other hand, a significant association had been reported between the rugae form and ethnicity which is considered a potential method for victim identification by forensic odontologists [93].

#### 3.4.2. Intercanine Width

In forensic odontology, the mandibular canines exhibit the greatest dimorphism between females and males [94]. These maxillary canines have several characteristics that make them a proper candidate for identifying the sexuality of victims. These teeth are less engaged with periodontal diseases, plaque, and calculus and abrasion by brushing. Above all, they are the least exposed to extraction due to aging [95].

Despite the importance of every method of identification, because of the nature of mass disasters, the methods based on hard tissues may be a more reliable way than the techniques based on soft tissues. For this reason, the scans and radiographs are used more in forensic dentistry, and the presence of PM photographs may be less useful than radiographs. It is clear that special restorations and the presence of AM dental casts are so valuable and can increase the accuracy of identification.

## 4. Medical Biotechnology Techniques

**Figure 5:**
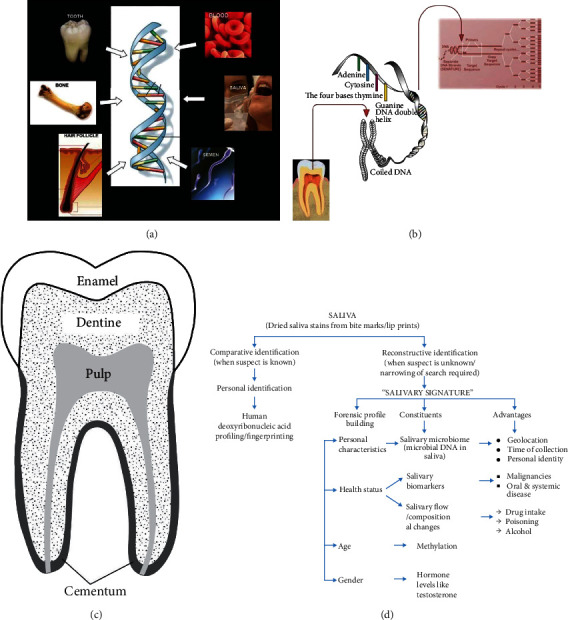
Sources of DNA for forensic analysis (a) (reprinted with permission, *Wolters Kluwer-Medknow*) [96]. Schematic photograph showing replication of DNA by PCR (b) (reprinted with permission, *Wolters Kluwer-Medknow*) [101]. A diagrammatic showing a human molar including various regions and tissues (c) (reprinted with permission, *Springer Nature*) [104]. The figure depicts the role of saliva in forensic identification, both comparative and reconstructive. It further explains the role of a salivary signature comprising of the salivary microbiome, biomarkers, flow, and composition changes in the generation of the biological profile of individual characteristics (d) (reprinted with permission, *Wolters Kluwer-Medknow*) [117].

An important identifier that is increasingly extensively used today is DNA fingerprinting, DNA profiling, or gene typing which includes extracting sets of codes encrypting the DNA configuration of an individual [2]. DNA fingerprint of every person is as unique as their fingerprint and much more precise. The DNA samples are obtained from the remnants in the crime scenes, victims, suspects, and/or inanimate objects around them (Figure 5(a)) [96]. In cases of lacking antemortem records and the poor state of corpses' preservation, DNA testing is applied [97]. In criminal and missing person cases or MS tragedies, DNA can also be obtained from the human dental remains for being used in DNA typing [98]. The sequence of tooth-extracted DNA from the unidentified subject can be then matched with known antemortem DNA samples such as stored blood, toothbrush, hairbrush, clothing, cervical smear, biopsy, or isolated samples from a parent or sibling to identify the unknown person [99]. Dental DNA samples can be better preserved and undergo more successful typing compared to other bone-extracted DNA samples (e.g., from long bones with thick cortical tissue like femurs) because they are highly protected in the enamel [100].

### 4.1. Techniques of Identification

The individual-specific sequence used for gene typing includes the polymorphic repetitive DNA that constitutes 20-30% of the noncoding DNA or junk DNA which contains >95% of the whole genome, while the protein-coding segments of DNA (genes) contain only 2-5% of entire cellular DNA and are highly preserved among species. Several functions have been hypothesized for the noncoding DNA including spacer in a single copy. On the other hand, the repetitive sequence appears as LTR or midi satellites, STR or mini satellites, and interspersed repetitive sequences (Figure 5(b)) [101]. There are three main methods of DNA fingerprints; (1) RFLP, (2) VNTR, and (3) STR or simple sequence repeat (SSR). Autosomal STR genotyping is the most popular one but, RFLP and VNTR do not require much amount of DNA. This is a valuable feature since DNA fragments found from the forensic scene are usually extremely scarce for being analyzed and too long for being amplified by PCR. Then, STR analysis is applicable with short sequences of DNA, much easier to be handled by PCR, and less time-consuming because it does not require the probe-hybridization [102].

Dental DNA can also be used for evaluating the age based on DNA methylation and estimating the biogeographical ancestry based on the sequence of the mtDNA and Y-Haplotype. These assessments provide essential information in forensic investigations. DNA methylation is an age-associated modification providing a promising biomarker with relatively acceptable accuracy for forensic chronological age estimation showing a mean absolute deviation of only 3-5 years [103]. In comparison with nuclear STRs, mtDNA is comparable to more distant relatives; then, it is better applicable in cases of missing persons. The analysis of mtDNA is also valuable and helpful in cases of obtaining little or no nuclear DNA from the crime scene [104]. In cases of analyzing relationships among multiple male contributors, the Y chromosome is especially useful because it is only inherited by men directly from their fathers [99]. However, a problem associated with using tooth-extracted DNA is the coextraction of calcium and collagen especially from the enamel tissue [104]. Another problem can occur due to environmental contaminants such as humic acid, fulvic acid, and metals. Also, microorganisms can negatively affect DNA purity during extraction and amplification processes [105]. DNA extraction methods include tooth- and saliva-related approaches. The most important recent studies in the field of sex and age estimation, as well as DNA dental fingerprinting, are summarized in Table 2. The release of endogenous intracellular nucleases with the beginning of the postmortem phase causes decomposing DNA. But DNA contained in teeth is highly preserved against the enzymatic degradation with the naturally hard mineral and low porous tissue of the tooth. Generally, the tooth is constructed from three parts of cementum, dentin, and enamel (Figure 5(c)) [104]. However, the exact anatomy of each tooth is different which is important to know to achieve the maximum DNA yield in studies. For example, it will help to know that the palatine upper and the distal lower molars or the most subsequent have the widest root canal, and the canine tooth has the longest canal within the same arch. Then, these teeth are the best sources for DNA extraction [106]. DNA molecules are obtained from almost all parts of the teeth except the enamel [107].

The dental pulp in the radicular and coronal portion of the teeth is the oldest source for DNA odontological forensic investigations [107–109]. Containing a great number of odontoblasts, fibroblasts, endothelial cells, undifferentiated mesenchymal cells, and nucleated cells of the blood, pulpal tissue provides a favorable amount of DNA to be isolated [2]. Therefore, in an intact fresh tooth, the dental pulp is considered the best source of DNA [110]. Another preferred source for DNA extraction is dentine which is a hard dense bony tissue protected by cementum and enamel [111]. Dentine is generated from odontoblasts present in the peripheral layer in a columnar form that is extended toward the thickness of the dentin [112]. AAR is also an accredited method for age estimation using dentine based on the nonenzymatic covalent modification of proteins [113]. Raman microspectrometry is another modern, highly selective, and noninvasive technology for age estimation that provides the chemical structure of a molecular fingerprint [114]. Considering that the DNA content of pulp can be negatively impacted by dental diseases, aging, and postmortem cellular degradation, some studies have sought other sources of nuclear DNA, especially in moist environments. Related publications explain that extracted DNA from different dental tissues is variable in quality and prioritize cementum for its less vulnerability to dental diseases or raising chronological age. Also, these reports hypothesize an important role for cellular cementum in the adaptation to occlusion and tooth movement after the eruption [110]. Also, the mitochondrial and nuclear DNA obtained from the tooth may reduce in quality and quantity due to the chronological age and dental disease and show variable efficiencies for STR typing. Also, while dentine-recovered DNA highly depends on the presence/absence of pulp, both nuclear and mitochondrial DNA can be finely extracted from the cementum especially when teeth remnants are degraded or ancient [104]. Large DNA strands with high molecular weight are commonly collected from the oral fluid at crime scenes from bite marks, cigarette butts, postage stamps, envelopes, etc. [115]. Compared to the blood as a source of DNA, saliva benefits from technical advantages such as easier and less invasive collection as well as no religious conflict, especially in infant subjects, children, and elderly subjects, and does not have the challenges [116]. Using modern technologies, only 0.1 ml of saliva samples are sufficient for obtaining enough applicable DNA [115]. The chronological age is detected from the level of salivary DNA methylation, and gender identification is exerted via measuring the salivary content of testosterone, whole saliva flow rates, etc. [117]. The individual characteristics evoked from the left salivary traces that can present age, gender, personal data, and health status are used for creating one's salivary signature (Figure 5(d)) [117]. In a comparative analysis by Watanabe et al., the sensitivity and stability of RNA-based and amylase-based markers were examined under different storage conditions and the RNA method was suggested as a supplementary method for the conventional amylase-based identification method [118]. Age, gender, and race are also determined by protein profiling and evaluating the total salivary protein concentration using the standard baseline of the protein variations [119]. Screening the *α*-amylase activity is a sensitive, simple, and cost-effective method for indicating the saliva presence; however, it is low specific [120]. Then, preserving the *α*-amylase stability is a must for catalytic and immunological forensic saliva investigations [121]. Different eating patterns, oral hygiene measures, humidity, climate, temperature, or even disease outbreaks can be used for recognizing various geographical locations since any locales result in a different salivary microbial community in composition and function [117]. Methods based on various oral resident bacteria such as *Streptococcus salivarius*, *Streptococcus mutans*, and *Veillonella atypica* are more specific than protein-based methods [120]. One of the most important species in forensic microbiology is *S. mutans* which is substituted in the mouth during birth and remain there throughout life. The most frequent genotyping analysis for oral microbial species is PCR-restriction fragment length polymorphism [122]. Saliva is routinely used as roadside testing for detecting the level of ethanol or psychotropic drug (e.g., cannabis) abuse. Corresponding THC is detectable in saliva due to smoking cocaine, while its secretion from serum to the oral cavity through saliva takes approximately 10 h [122].

DNA extraction could be one of the most valuable techniques among all methods of human identification, but the sensitivity of techniques, high prices, and time-consuming methods are its undeniable weakness.

## 5. Lip Print

The soft tissues of lips with their wrinkles and grooves on the labial mucosa provide applicable evidence for personal identification and criminal investigation called lip print [123, 124]. The study of the individually unique characteristic pattern of the sulci labiorum, similar to the fingerprints, is called cheiloscopy [124, 125]. Some information about the contributors in a crime scene such as the number of the people, their genders, cosmetics used, and the pathological transformations of lips can be figured out from the lip prints [126]. The advantage of lip prints is that they can be recognized conveniently since they recover after trauma, inflammation, and diseases such as herpes [127]; however, its pattern may change due to pathology, postsurgical alteration, or loss of support due to loss of anterior teeth [128]. The grooves of the lip also change based on the open/close status of the mouth since they can be well-defined or ill-defined in close and open states, respectively. Legibly, they are difficult to interpret in the open position [128]. Although some studies implicate certain lip groove patterns to be related to each of the sexes or geographically distinct populations, this specificity is not completely proved [129]. Recent identification researches using lip prints are summarized in Table 3.

### 5.1. History and Classification

Initially, cheiloscopy (Cheilo means lips in Greek) was noted by anthropologists, and Fischer was the one who described this epithet in 1902 [130]. Three decades later, criminologists such as French Edmond Locard started to use the trace of lip furrows for human identification [131, 132]. Afterward, lip prints are classified based on their consistent groove patterns which resist many afflictions [132, 133]. Several lip print classifications have been established among which the most popular is the one made by Suzuki and Tsuchihashi [10, 126, 134, 135]. According to their classification, lips are categorized in four groups: (1) thin lips that are frequent in European Caucasian, (2) medium lips that are the most frequent type, (3) thick or very thick lips that are commonly seen in African Americans and usually associated with a lip cord inversion, and (4) mix lips which are often seen in Orientals [131].

### 5.2. Methods of Identification

**Figure 6:**
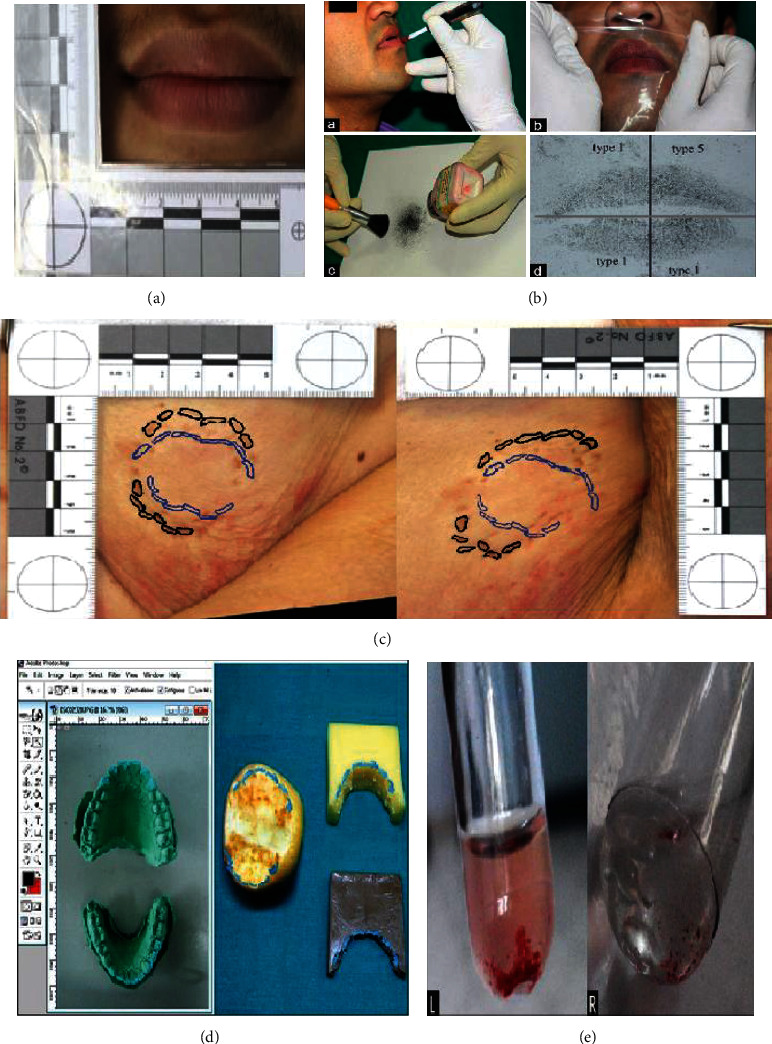
Photography technique of taking lip print (a) (reprinted with permission, *Hindawi*) [138]. (a) Application of persistent lipstick with the applicator brush, (b) lifting the latent lip print with cellophane sheet, (c) application of lysochrome dye powder with a round brush, and (d) final lip print after digitization and division into quadrants (b) (reprinted with permission, *Wolters Kluwer-Medknow*) [145]. Any alteration in the appearance of bite marks depends upon the body position. The bite was inflicted with the arm straight at the side (left). The bite mark (black) and biter's overlay (blue). Change in bite pattern due to arm positioned over the head [158] (c) (reprinted with permission, *Oxford University Press*) [158]. The selection of incisal edges using the “magic wand” wizard tool in Adobe Photoshop software from the photograph of the dental cast and the superimposition of computer-generated overlays over the photograph of a bite mark on apple, cheese, and chocolate to check for matching (d) (reprinted with permission, *Wolters Kluwer-Medknow*) [159]. Macroscopic examination of the agglutination in dentin (left) and pulp [158] samples (e) (reprinted with permission, *Wolters Kluwer-Medknow*) [163].

The variety of lip print analysis methods has brought up an inconsistency in the related literature all over the world [136]. Among these various methods are photographing, direct prints onto paper, and generating lips 3D casts which finely display the lips groves using dental impression materials [137]. Comparison methods for lip prints (e.g., picture/lens magnification) have been facilitated using a computer [129]. For utilizing digital methods, clear lip print photographs in a standardized position are required (Figure 6(a)) [138]. Different substances including aluminum powder, silver metallic powder, silver nitrate powder, plumb carbonate powder, fat black aniline dyer, cobalt oxide, lysochrome dye, or fluorescent dyes can be used for visualization of the latent lip prints [139]. Aluminum and magnetic powder are the most common substances usually used for identifying and tracing lip prints left in crime scenes [140]. The lipsticks or cellophane tapes are usually used for manual generating lip print traces in scientific studies [125, 127, 141–143]. Lipsticks contain several compounds, oils, or waxes in their complex constitution [132]. In cases of similar colors of lip print and its background or the trace being old, fluorescent agents are used for visualizing the lip's traces [144]. Furthermore, a study by Ramakrishnan et al. has suggested using persistent lipstick, cellophane sheets, and lysochrome dyes for sex determination (Figure 6(b)). They also maintained the latent lip prints in a digital database [145]. Another adjuvant tool employing lip prints in forensic investigations is lip outline patterns. Maloth et al. have shown that lip outline patterns have the potential to be used for human identification since they are individually unique [146].

## 6. Bite Mark

An arguable area of forensic odontology which is mostly applicable in homicide, rape, sexual assault, robbery, and child abuse criminal cases is bite mark analysis [35, 147]. Although the bite marks are unique to individuals even in identical twins, accepting bite mark evidence in courts needs fundamental validation and high scrutiny investigations to ensure its reliability [148, 149]. Various scientific principles and factors are required to be considered to make the bite mark applicable for personal identification. The injury site, size, and age, as well as the skin mobility, the degree of trauma, and the state of structures underlying the skin in the injured area are some factors that need to be considered [150]. Many studies impact the more accuracy and reproducibility of bite marks obtained on food items than those on the skin [151].

Human bite marks are usually left by the incisors, canines, and premolars that create two opposing U-shaped arches. The open spaces between U shapes may contain hematoma due to soft tissue compression. The most prominent marks are created by maxillary canines with a normal distance of 25–40 mm [152]. Recent human identification researches of bite marks are summarized in Table 3.

### 6.1. Bite Mark Types

From a forensic odontology viewpoint, teeth marks comprise three main types: (1) bite marks on comestibles, (2) bite marks on the assailant body due to victim's self-defense, and (3) bite marks left on an assault or murder victim's body usually in cases of sexual harassments which are mostly found on the breast, neck, or cheek [153]. The injuries caused by the human bite are divided into two categories based on the force they have applied on the skin to lose its integrity (closed fist injury or fight bite) or to breach and probably avulse the tissue (occlusive bite injury) [154].

### 6.2. Methods of Identification

A comprehensive description of reliable methods of assessing the bite marks is available on the latest Manual published by the ASFO, in the section titled “Bite Mark Pattern Recognition and Collection from Humans and Inanimate Objects: Non-Invasive Analysis” [155]. According to this manual, several techniques can be used to analyze the bite mark patterns. One of the most accurate models for identifying human bite marks is dental casts. Also, registering the bite marks of volunteers on the clay, wax sheet, styrofoam sheet, and human skin by overlay is recommended by the American Board of Forensic Odontology [148]. Overlays are obtained by hand tracing, xerographic images, or through X-ray films. Then, the impressions generated by these life-sized overlays are surveyed by comparing with the bite mark evidence from the crime scene or the suspect's teeth [156]. Cone Beam Computed Tomography is another suggested forensic technique especially for analyzing the bite marks in foodstuffs [157]. The position of the body can cause differences in the bite mark appearance (Figure 6(c)) [158]. An indirect technique has been applied by Daniel and Pazhani for victim identification using computer-assisted overlay generation. In this method, life-size photographs of dental casts are used for generating overlays from anterior dentition. For this purpose, the “magic wand” wizard tool in Adobe Photoshop CS4 software is applied (Figure 6(d)). An adequate matching between these overlays and the real bite mark pattern obtained from the physical evidence (such as foodstuff) can be met by superimposing one over the other in various angles [159]. Computer-generated overlays are the most popular and reliable method of overlay generation [160].

Lip print and bite marks are practical in forensic science. However, as they are soft tissue-related methods, in disasters with serious facial damage or fires, they are useless and human identification needs more precise methods which are markedly in relation with hard tissues like bone and teeth along with approaches based on scanning radiographs.

## 7. Blood Group

Since decades ago, the ABO blood categorization methodology has been considered a reliable medicolegal identification system based on the antigen-antibody reactions of every individual's RBCs membrane and remains unchanged throughout life [161]. Based on the ABO system, people are categorized into groups of A, B, AB, or O blood groups [70]. There are enough ABO antigens in dental tissues to be used for identifying even highly decomposed bodies [162]. Another combined antigen used in ABO system identification is the Rh [163]. There are also other antigens and blood group systems, but they are not as applicable as the ABO and Rh groups in practice, since they are weak or the corresponding antibodies are not conveniently available [164]. Recent blood group applications in forensic researches and human identification studies are summarized in Table 3.

### 7.1. Teeth-Related Diagnosis

Although pulp soft tissue is all surrounded by dental hard tissues, the ABO antigens diffused from both blood and saliva can be isolated from the tooth pulp since it contains a lot of blood vessels [2, 163, 165]. However, the distribution of these factors gradually decreases from the pulp cavity wall toward the enamel and dentin edge [2]. ABO factors are also found in dentinal tubules [163]. Siracusa (1923) developed the AE technique firstly, and Kind (1960) modified his method. However, later on, more modifications have been applied to improve AE sensitivity, specificity, and resistance to external interfering agents. AE is extensively used for detecting the blood group from various forensic evidence such as dried stains, tissues, secretions, and teeth. An important pro of this method is the reusability of its prepared and processed antigenic material [166]. More recently, Kumar et al. have suggested a validated method for obtaining red cell agglutination from the dental samples (Figure 6(e)) [163]. Furthermore, there are other methods such as absorption elusion, hemagglutination, PCR, and histochemical techniques for determining the ABO/Rh group. Among all, PCR shows high sensitivity and specificity putting it at the highest priority [162].

### 7.2. Saliva-Related Diagnosis

As mentioned before, water-soluble antigens are hypothesized to be infused from the saliva to the tooth tissue (infusion sedimentation theory) [167]. Therefore, several studies have been allocated to developing new techniques for detecting ABO factors from saliva with 100% accuracy. Two main methods currently established for this aim include the AI method and the absorption-elution method or AE which is easier and simpler [164]. SPR imaging is another ABO detecting method based on the interactions between immobilized biomolecules and DNA-protein or cells in the solution phase. SPR imaging has been used to indicate another blood categorizing type called ABH system with 100% accuracy [70].

The ABO and Rh groups can raise the accuracy of forensic human identification. Since pulp tissue is protected by tooth outer layers, detection of these groups may be done more precisely in comparison with saliva or a drop of blood which are vulnerable to environmental contamination. So, it is another reason why the presence of only a single tooth can help dramatically during various identification methods.

## 8. Conclusion

Forensic dentistry or odontology is attracting an increasing concern for its importance and efficiency in identifying victims of different tragedies and mass disasters. This article is aimed at having a comprehensive survey on the most significant aspects of forensic dentistry including dental radiographs and scanning, sex and age estimation, medical-biological techniques, blood grouping, lip print, and bite mark identification. This review attempted to highlight the conventional and new approaches of forensic odontology in each aforementioned part. From our standpoints, because of the ease of use, the velocity of techniques, and being cost-benefit, the role of dental radiographs and scans are more effective in comparison with others in oral-and-maxillofacial related approaches of human identification. The reason for that is they illustrate resistant-to-disasters hard tissues in a facial complex like bone and teeth and they act faster than histologic approaches along with being more economical. Two key factors are required for efficiently applying dental records in human identification. These factors include regular oral health follow-ups and preserving high-quality dental records in the form of dental charts, radiographs, photographs, impressions, casts, etc. Different patterns and anatomy of teeth, jawbones, and sinuses (including the missing, filled, and decayed teeth) also increase the specificity of dental identification methods. Usage of other characteristics such as lip print patterns, bite marks, oral microbiome, and salivary biomarker databanks, and ABO blood groups of individuals can help radiographs and scanning methods to reach more precise detection. This is clear that each method has its limitation. The scanning approaches are highly based on the quality of imagination, magnification, the accuracy of measurements, and the correct interpretation of results. On the other hand, soft tissue-related methods are under the influence of the severity of the disaster, the situation of the PM environment, and the accuracy of the histopathologic analysis. We hope that our attempt in extracting maximum information about forensic dentistry will be beneficial to society in human identification.

## 9. Future Direction

Ten years ago when the new technology of CT had been accepted to be routinely used for human identification purposes, the 3D surface comparison had just emerged. Today, the 3D surface comparison is the main technique for gender determination. We expect further development and rapidly advancing forensic odontology technologies related to imagery such as CT and CBCT. Also, 3D datasets including CT and AM 3D surface scan data are expected to play an important role in applying forensic odontology in DVI. From our standpoints, 3D approaches like CBCTs are the future of DVI because they provide valuable information. The reason why they are so important is the fact that not only would be the presence of teeth variations clear in advance techniques but also various types of significant radiographs like OPGs, lateral and PA cephalometric can be extracted in one CBCT and precise measurements can be accomplished easier in these practical modes of digital scans. 3D AM dental profile and PM virtual models can be prepared using 3D intraoral scanners becoming an indispensable toolset for forensic investigations. Like any other field, statistical and computational analyses, as well as modeling techniques, are extensively used in designing and implementing researches about identification methods. In the same regard, digital forensics as an integral part of forensic researches has considerably reduced the costs of technology and increased the accuracy of forensic investigations. Also, advances in molecular biology technologies have helped more efficient DNA extraction from less available material even under adverse conditions. Forensic biorobots for DNA extraction, laser microetching for labeling of metallic prostheses, Raman spectroscopic analysis of dentin for age estimation, forensic thanatology for investigation of every phenomenon related to death, virtual autopsy, intraoral scanners for improving the accuracy of bite mark impressions, and retouched images for fraudulent purposes are some advanced tools and methods used in forensic investigations. However, as always, conventional methods hands in hands with advanced technologies exert more convenient and accurate results. It is wise to mention that using an interdisciplinary approach using both forensic medicine, forensic dentistry with the emphasis on advanced scanning technology will enhance the accuracy of clarifying a questionable identity in a legal jurisdiction.

## Figures and Tables

**Table 1:** Dental records.

Type	Method	Outcomes	Ref/year
Prosthodontic view	A systematic review on applying prosthodontic for forensic odontological aims.	The identification process in accidents and disasters is accelerated using marked dental prostheses.	[86]/2012
Rugae pattern	The individuality of the rugae pattern was assessed by Martin dos Santos' classification.	Rugae pattern was unique in each subject including dizygotic twins, showing no symmetry in neither number nor distribution.	[89]/2012
Dental charts	Basic dental characteristics were coded into letters and analyzed by a specifically written computer program.	The diversity of dental patterns can be efficient for human identification.	[66]/2013
AM/PM endodontic treatment records	An unknown body was identified by comparing AM and PM evidence of endodontic treatments.	Dental radiography records including endodontics are reliable legal tools in forensic dentistry.	[8]/2014
Multislice CT (MSCT)	AM/PM comparison of both teeth and bone imaging data.	MSCT added a new dimension to the specialty of forensic radiology.	[69]/2014
Palatal rugae patterns	Predominant shapes of rugae patterns were statistically analyzed and categorized in both genders.	The most predominant type of palatal rugae pattern was the “Wavy” variant in both genders.	[91]/2014
Radiography	A precise comparison of AM and PM radiographs.	Comparable radiographs are shown to be essential evidence for personal identification in MD.	[74]/2015
Frontal sinus pattern matching	Personal identification was conducted using frontal sinus radiography matching.	This method is shown to be useful in personal identification cases in the absence of other methods.	[78]/2015
DNA analysis in combination with several other records	Several AM dental records including dental prosthesis, restorations, crowns, and bridge were used with or without DNA analysis.	Forensic odontology in combination with DNA analysis could accurately identify 97.4% of victims.	[65]/2015
Ideal dental record form	The records from private clinics and academic teaching hospitals were analyzed in a comparative cross-sectional study.	The knowledge of the medicolegal importance of dental record maintenance increased among students.	[63]/2015
AM and PM data	A forensic identification case was conducted using three comparative techniques for analyzing the dental traits from a single smile photograph.	A charred body was positively identified using the AM and PM records based on a smiling photograph.	[84]/2015
Palatal rugoscopy	Palatal rugae pattern, incisive papillae shape, median palatal raphae length, and dental arches shape were analyzed.	Palate traits were individually for both genders.	[90]/2015
Orthopantomograms	Nine types were determined for the full dentition, maxilla, and mandible patterns, and their diversity was studied in dental radiography.	Orthopantomograms are shown to be reliable tools for victim identification.	[73]/2015
The responses of crime scene investigation (CSI) officers to the questionnaire	The designed questionnaire assessed the awareness and knowledge of CSI officers on forensic odontology.	The police personnel needs to be educated about the necessity of forensic dentistry.	[68]/2016
Palatal rugae dimensions obtained with alginate impressions	Dimensions of the palatal rugae were measured on the rugae patterns traced on the dental casts using a digital caliper and compared between two tribes.	Karnataka and Kerala individuals showed a significant difference in the dimensions of the palatal rugae.	[93]/2016
Radiographic endodontic records	A comparative dental identification was conducted using the periapical radiographs reproduced by imaging acquisition techniques.	These comparative techniques could positively identify all victims based on their dental morphology and treatment intervention.	[67]2016
Dental prosthetics identification (DPid)	A digital database was established containing patient information accessible for dentists, laboratory technicians, and patients with different security levels.	DPid was suggested as a qualified tool for solving forensic cases independent from the DNA exam.	[87]/2017
Dental records in a military population	A forensic dental symbols® system was designed to collect the information in a generic codification (unrestored, restored, missing, and crowned teeth).	Quality dental records were proved to be required as mandatorily stored and be easily accessible in all countries for dental identification forensic.	[4]/2017
Extraoral dental radiography	The lateral oblique radiographs of left and right posterior teeth and the contact radiograph of anterior teeth were obtained. The scattered X-ray dose of each in the resolution test was calculated by the ionization chamber-type survey meter.	This method is especially useful for dental identification of disaster victims or patients who have problems in opening their mouths.	[6]/2017
Maxillary canine index and maxillary first molar dimensions	The maxillary first molar dimensions (buccolingual and mesiodistal), maxillary canine index (mesiodistal), and the intercanine distance were measured on the cast using a vernier caliper.	The maxillary first molar BL dimension is shown to be the most reliable indicator trait for gender determination.	[88]/2017
Rugae shape and positional changes	Pre- and postmaxillary expansion casts of palatal rugae were assayed for the shape of rugae, and the distance between the median points and lateral points of the first and the last two rugae on both sides of the mid-palatal raphe were measured.	The palatal separation can be quantified after the expansion of the maxillary arch using the interrugae distance.	[92]/2018
The responses of commissioners to a questionnaire	A questionnaire on using dental evidence in human identification was designed and distributed in a commissionerate, and response data was interpreted.	The findings of this survey showed that dental professionals and law enforcement agencies need to work in close association.	[1]/2018
Dental records, molecular traits, and identity of deceased persons	Describe the benefits of a multidisciplinary approach of human identification using forensic odontology and molecular biology/biochemistry together.	Such a multidisciplinary approach results in an adequately reliable identification outcome.	[14]/2018
Forensic photography	Systematically review the various aspects, diverse applications, and recent advancements of forensic photography.	Forensic photography was introduced as a crucial tool in forensic dentistry from both mechanics and technique aspects.	[81]/2018
Humanitarian forensic	Survey the potentials of forensic dentistry aimed at identity investigations in cases of preventing human rights violations.	Teeth and jaws are greatly helpful in providing the required data for disaster victim identification (DVI).	[62]/2019
A dental remnant from disaster	The AM and PM radiographs, computerized tomography (CT) data, and 3D scan data were used.	3D dataset comparison is inferred to be the future of forensic dentistry DVI techniques.	[15]/2019
Posteroanterior (PA) skull radiographs	The frontal sinus digital photographs were transferred to Adobe® CS4, and the frontal sinus dimensions were measured.	The asymmetric and individual morphology of the frontal sinus makes it an effective identifier in forensic anthropology.	[37]/2019
Panoramic images	Four oral and maxillofacial radiologists and four dentists who were not oral and maxillofacial radiologists were recruited to match the image pairs depicting a patient and qualitatively rate each match and indicate their used anatomical structure.	Panoramic images are qualified tools for identifying patients lacking teeth.	[76]/2019
Dental pattern with adopting chronology of dental treatment	AM and PM orthopantomographs were analyzed for the dental pattern.	The automatized version of this method was introduced as more efficient and comprehensive.	[7]/2019
3D dental models and intraoral scans	A 3D dental identification system was developed using iterative closest point (ICP) and principal component analysis (PCA) using 3D dental models and intraoral scans [158].	Automated identification from dental data (AutoIDD) could accurately identify the matches and differentiate the matches from nonmatches.	[11]/2020
Dental panoramic radiographs	AM and PM DPR were compared by computer vision.	This method was efficient for the identification of dental traits even if they are added or removed in the past.	[75]/2020

**Table 2:** Sex and age estimation and dental DNA fingerprinting.

Type	Method	Outcomes	Ref/year
*Sex estimation*			
Foramen and perpendiculars	The distances from the mental foramen to the lower border of the mandible were measured by drawing tangents to the superior and inferior borders of the foramen and perpendiculars from the tangents to the lower border of the mandible.	This parameter showed adequate gender dimorphism in the north Indian population.	[45]/2013
Frontal sinus	The right and left areas and maximum height and width of the frontal sinus were measured using the Caldwell view.	The findings represented no specific diversity between genders in the size of the frontal sinus and logistic regression.	[36]/2014
Maxillary landmarks	Intercanine width, interpupillary distance, intercanthal distance, and interalar distance were measured.	Intercanine width displayed association with different reference points in the faciomaxillary region and inter-gender variation.	[95]/2015
Barr body	Barr bodies were analyzed in nucleated cells from dental pulp using light and fluorescent microscopy.	Barr body is proved to be a reliable identifier for sex identification in forensic dentistry.	[57]/2015
Digital orthopantomograph	Linear and angular measurements of selected radiographic and tomographic images were conducted using the KLONK image measurement software tool.	Both radiographic and tomographic images were suggested as useful tools for sex prediction in forensic dentistry.	[72]/2015
Human maxillary sinuses	The height and width of the right and left maxillary sinuses were measured using the software ImageJ 1.47v.	The height and width of the maxillary sinuses are shown to be highly discriminative between the two genders.	[32]/2016
Mandibular radiographs	Ten variables of the mandibular were evaluated using the Planmeca Romexis software.	Except for the gonial angle, all other nine mandibular variables are shown to be reliable gender identifiers in South Indians.	[71]/2016
Mandible	A systematic review was conducted using the related literature available at MEDLINE, PubMed, and EBSCOhost.	75% of the total of 20 extracted studies showed a positive correlation between mandibular parameters and gender dimorphism.	[43]/2016
Panoramic radiographs	The height of the mandible and the distance from the superior border of mental foramen and the inferior border of the mental foramen to the lower border of the mandible were measured.	The radiographic analysis of mental foramen showed gender dimorphism in the Maharashtra population.	[44]/2016
Lateral cephalometric radiograph	Lateral cephalograms in standard position with centric closed teeth and relaxed lips were impressed, and the cephalometric traits were sized by a digital caliper.	Findings showed a significant sexual dimorphism in the skull since 6.5 years old.	[77]/2016
Maxillary sinus CT images	The mediolateral, superoinferior, anteroposterior, and maxillary sinus measurements were calculated.	The maxillary sinus dimensions can be used for fairly accurate sex estimation.	[33]/2016
Orthopantomographs	Panoramic graphs were used for measuring maximum ramus height, bigonion width, and bicondylar breadth.	These measures represented reliable parameters for predicting the gender of the deceased person.	[9]/2017
MRI of maxillary sinus	MRI data were collected from the maxillary/paranasal sinuses, and the volume of the maxillary sinus was measured.	Measurements of maxillary sinuses using MRI are shown to be a potential identifier for sex estimation.	[30]/2017
Odontological sex estimation	A systematic review was conducted using grey literature and databases of MEDLINE, PubMed, Cochrane, SciELO, and LILACS.	Numerous studies emphasized the possibility and importance of sex estimation using dental evidence during human identification processes.	[16]/2017
Longitudinal tooth sections	Longitudinal ground sections of teeth in the buccolingual plane along the midline were prepared, and the pulp was removed.	These traits were introduced as a rather reliable tool for sex determination in teeth being extracted over 6 weeks. Additionally, it can be used for determining the ABO blood group.	[167]/2017
Maxillary sinus CT images	Tomographs were used to evaluate the size of the maxillary sinuses, as well as the mediolateral, superoinferior, and anteroposterior dimensions.	These parameters in CT images could be applied for gender determination.	[29]/2017
Gender determination	Two observers recorded the lip prints, mandibular canine index, and facial index measurements.	No significant difference was observed in odontometric analysis which needs a larger study community to validate these results.	[19]/2018
Frontal sinus radiographs	Digital radiography and morphometric evaluation of the frontal sinus were done by Photoshop.	The radiomorphologic method was useful for gender estimation in the Saudi population.	[34]/2019
Mandibular measurement	Morphometric measures of mandibular were estimated from the angles formed at different locations.	The angle of the intersected lines from the left and right gonion to menton (Gn-M0) of mandibular can be considered a sex indicator.	[41]/2019
*Age estimation*			
Radiological methods	Systematically review 46 articles on using dental radiological methods for age identification.	The radiographic method was discussed to be a simple and low-cost method for age estimation in comparison with histological and biochemical methods.	[23]/2011
Dentin translucency	Translucency measurements obtained from a digital method and caliper were compared.	The two methods of translucency measurements showed no significant difference.	[53]/2013
Relationship of chronological age and dental age	Panoramic radiographs were included, and various parameters were measured, and the result was analyzed	The accuracy of this method correlates with the precision of evaluations, quality, and number of OPGs.	[22]/2013
Lower third molar radiographies	The mineralization of third molars was measured by assessing the visibility of the periodontal ligament.	This technique was useful for determining people elder than 21 in the Portuguese population.	[26]/2014
Human cementum and secondary dentin	The longitudinal ground sections of the extracted teeth were prepared, and the cemental/incremental lines and thickness of secondary dentin were calculated.	Cementum annuli quantification and secondary dentin amount were suggested as reliable methods for age estimation in human identification.	[51]/2014
Maxillary landmarks	The intercanine width and distances of pupillages, canthi, and interalar were measured.	Intercanine width showed the most correlation with the chronological age of subjects.	[95]/2015
Dental panoramic tomograms	The reliability of Demirjian and Indian formulas for age estimation was calculated.	Both Indian and Demirjian formulas showed underestimation of age. Then, population-specific formulas are suggested to be developed based on the ethnic and environmental variations.	[27]/2015
Digital orthopantomograph	Systematically review related articles, and the KLONK image measurement software tool was selected for calculating the linear and angular measurements of radiographic images.	Radiographic and tomographic images were discussed to be an essential tool for the prediction of age in human identification forensic processes.	[72]/2015
Morphometric radiographs	The radiographs were scanned, standardized to the normal size of radiographic film, and the obtained morphometric measurements were compared.	Total pulp area negatively correlated with age.	[56]/2015
Dentin translucency	Translucency measurements obtained from the digital method and caliper were compared.	The two methods showed identical efficiency in providing translucency measurements.	[52]/2015
Cervical vertebra maturation	A computer App (Cephalometar HF V1) was created and used to label the contours of the cervical vertebrae 2-4 on the digital lateral cephalograms.	This App was discussed to be a reliable method for estimating the cervical vertebral maturation stage.	[40]/2015
Third molar panoramic radiographs	The correlation between the third molar development stage and actual age was analyzed.	The third molar calcification level was introduced as a chronological age indicator.	[17]/2015
Cemental annulations	Cemental lines and the eruption age of teeth were added to obtain the chronological age.	The middle third of the tooth root is propounded to be the most suitable part for calculating annulations.	[47]/2015
Longitudinally ground sections of teeth	The thickness of the cementum was measured using a light microscope and a micrometer eyepiece.	Measuring the cementum thickness at the apical one-third of the root and the cementum overlap or coronal migration at the cementoenamel junction (CEJ) is applicable for forensic age estimation.	[49]/2016
Orthodontics records	The pre- and postorthodontics treatment records were compared.	Orthodontists are required to maintain the dental records in a proper situation.	[83]/2016
Mental foramen position	The foramen upper border-mandible inferior border distance was measured to evaluate the horizontal/vertical position of the mental foramen.	The mental foramen position showed high variability in the Lebanese population.	[46]/2017
Maxillary sinus MRI	Maxillary sinus dimensions were obtained from the MRI of the brain including the paranasal sinuses.	MRI was considered useful for measuring the traits of maxillary sinuses that support the age estimation.	[30]/2017
Cervical vertebra maturation stages	Dental age based on the developmental stages of upper and lower third molars was matched with the skeletal maturation based on the cervical vertebrae maturation stage.	Dental age showed a positive correlation with skeletal maturity in both genders.	[39]/2017
Coronal pulp radiographs	Estimate age using tooth coronal index (TCI) of mandibular first molar and second premolar teeth.	TCI of the pulp cavity was a precise, short, cheap, and noninvasive method used in the Indian population.	[61]/2017
Longitudinal teeth sections	Longitudinal ground sections of teeth in the buccolingual plane along the midline were prepared, and pulp was removed for age determination using cemental lines.	This method was represented as adequately reliable for determining the age, sex, and blood group not only in freshly extracted teeth but also in teeth extracted even after 6 weeks.	[167]/2017
Third molar	The chronological age of patients was evaluated using the Demirjian formula that showed the third molar development stage.	The G and H stages represented individuals above 18 and the E and F stages represented people under 18 in the Iranian population.	[25]/ 2017
Frontal sinus radiographies	Using a direct match of AM/PM MSCT, the morphological data including the lateral expansion of the left lobe, anteroposterior dimension, and the position of median and accessory septa of the sinuses were collected.	The finding confirmed the importance of storing and interpreting radiographic medical data for forensic radiology applications.	[79]/2017
Mandibular parameters	Radiographs of patients were selected to see superior and inferior aspects of the mental foramen and the ramus height.	The ramus height and the mental foramen can be used effectively in the identification of gender using digital panoramic radiography.	[42]/2018
Pulp volume (PV) and tooth volume (TV) measurements	The PV, TV, and PV/TV ratio was calculated for each tooth.	Despite gender, age had a strong correlation with the PV/TV ratio of especially maxillary central incisors.	[55]/2018
CT and MR imaging	Systematically review the literature on using CT or MRI for dental age determination.	The highest accuracy will be obtained when there is a combination of different teeth, methods, and disciplines.	[28]/2018
Cementochronology	Nine anthropological cases were taken from the Forensic Medicine Institute of Lille (France) and compared using routine osteological and dental methods.	The age estimation accuracy and precision of the cementochronological method were comparable to the traditional methods.	[48]/2018
Orocervical radiographic index	New age estimation equations were presented and verified through dental and cervical vertebrae examinations.	The new equations included the cervical vertebrae and dental data and provided high accuracy for age estimation.	[38]2018
Cementum photomicrographs	Formalin-preserved teeth were sectioned, and the annulation lines were counted.	Incremental lines of cementum are shown to be reliable identifiers of the chronological age.	[50]/2018
Paranasal sinuses CT images	An automated tool was developed for measuring the total and air-free volume of the maxillary sinus.	The presented tool is shown to be rapid, reliable, robust, accurate, reproducible, and extensively applicable in forensic dentistry.	[31]/2018
Anthropological parameters	Systematically review the application of recent advances in forensic methodologies including histology, taphonomic impact, anatomical, biochemical, and mathematical approaches.	Advanced technologies such as 3D imagery and scanning and especially biochemical analyses such as dry bone direct examination will impact the progress in the anthropological age estimation process.	[20]/2019
Frontal sinus radiographs	Photoshop was used for digital radiography and morphometric evaluation of the frontal sinus.	The radiomorphologic method was useful for age estimation in the Saudi population.	[34]/2019
TCI (tooth coronal index)	Premolar and molar orthopantomograms were digitally evaluated for the coronal height (CH) and the coronal pulp cavity height (CPCH.	TCI is shown to be a precise, noninvasive, simple, and reliable indicator for age estimation in both living and dead victims.	[54]/2019
Aspartic acid racemization (AAR)	The maxillary first premolar was powdered by heating for 0-72 h at 110°C for AAR analysis, and its AAR rate stability was examined during the storage time.	No significant changes were observed in the AAR rate stability suggesting the powdered dentin as a reliable sample for age estimation procedures.	[21]/2019
Dental sections	Teeth sections were prepared, and various parts of dentin were assessed using a Raman spectroscopy machine.	Raman microspectroscopic analysis of teeth was discussed to be applicable for age estimation.	[114]/2019
*Dental biomolecular methods*			
Dental DNA fingerprinting	Systematical reviewed the recent literature on using DNA fingerprinting in forensic investigations.	This new technology has made a revolution in the field of individual identification.	[101]/2010
Dental DNA extraction	The quantity of DNA obtained from the crushed teeth and removed pulp were compared by the standard method of trepanation and amplified DNA microsatellites.	The trepanation method provided more and better DNA for genetic profiling aims.	[112]/2010
DNA profiling in forensic dentistry	Related articles were extracted from the PubMed and Embase electronic databases from 1980 through July 2010.	Teeth were recommended for DNA analysis for their high-quality DNA content that can be helpful in all forensic investigations.	[99]/2011
Dental DNA extraction	Dental DNA was extracted at different times using multiple endodontic techniques by the Minifiler® kit.	The endodontic methodologies are shown to be simple and efficient for genetic analysis of dental DNA.	[107]/2011
DNA extracted from ancient skeletal remains	DNA was extracted and analyzed from two human skeletons from a medieval burial.	Dental DNA was of better quality for later analyses compared to DNA extracted from the bone.	[100]/2012
The literature on dental DNA remains	Summarize the available data on the DNA content of different parts of the tooth and the effect of postmortem changes on the quality of extracted DNA in different extraction protocols.	Some tooth selection and sampling methods were recommended to maximize the efficiency of DNA typing and genetic analysis for identification aims.	[104]/2013
Nuclear DNA from the cementum	The nuclear DNA from the cementum was examined, and its yield was quantified. Also, the effect of bleach on nuclear DNA was explored by histological and quantitative PCR methods.	Cementum was represented as a valuable and accessible source for extracting dental nuclear DNA.	[104]/2013
DNA extracted from oral fluid (OF)	Applications of OF-extracted DNA were reviewed.	OF provides a useful source for forensic dentistry.	[115]/2014
DNA extracted from saliva	The quantity and quality of extracted DNA were assessed using spectrophotometry and PCR.	Saliva was represented as a useful source of DNA for forensic purposes.	[116]/2014
DNA profiling	Review the evolution of DNA isolation and fingerprinting methods.	The individualized nature of DNA has made it acceptable evidence for the court in forensic cases.	[102]/2015
Human skeletal extracted DNA	Various nondestructive methods of DNA extraction were assessed to replace the pulverization method.	A method consisting of a cervical cut was purposed that improved the accessibility of the pulp cavity.	[106]/2015
Nuclear DNA profiling	The integrity of the nuclear DNA extracted from several hard tissues was evaluated using a short tandem repeat (STR) typing and compared.	Rib is shown to be a good source for DNA obtain.	[97]/2015
Complete DNA from caries teeth	Teeth were fragmented, complete DNA was extracted from the dental pulp, and DNA profiling was exerted by the AmpFlSTR® NGM SElect™ kit.	Caries teeth showed to be valid sources for DNA extraction as healthy teeth for forensic purposes.	[109]/2015
Extract DNA from teeth	Two pulverization methods of dentine roots were compared.	A minimally invasive method for extracting DNA from dentine root was suggested that could preserve the tooth and crown morphology.	[111]/2016
DNA extracted from dental pulp	Dog canines were used for total DNA extraction and sequencing by the next-generation sequencing [70]. The sequence analysis was conducted by blasting.	Dental pulp is shown to be a qualified source for DNA in both modern and ancient samples.	[108]/2017
RNA extracted from saliva	RNA extraction from saliva was compared to the amylase-based conventional methods.	Saliva-extracted RNA was introduced as a supplementary method for identification purposes.	[118]/2017
DNA extracted from teeth	Dental DNA was extracted from a submersed adipocere body and an exhumed body.	Cementum had preserved DNA from degradation.	[110]/2018
Salivary total protein	Salivary total protein concentration was profiled, and its changes were surveyed.	The total salivary protein is shown to be an applicable personal identifier in the Gujarat population.	[119]/2018
Salivary microbiome	A systematic review was performed on the related literature to the role of saliva in comparative and reconstructive identification on PubMed and Google Scholar databases.	Salivary microbiome and biomarkers could provide indicative data about the lifestyle, geolocation, salivary flow, etc. that can help forensic identification.	[117]/2018
Advanced glycation end products (AGEs)	The pentosidine content of root in both healthy, pink, carious, diabetic, and heated teeth as well as the extent of AAR was determined.	All these disturbing factors could impact the efficiency of both methods.	[113]/2018
Salivary *α*-amylase	The stability of salivary *α*-amylase was evaluated using qualitative and quantitative tests (RSID™-saliva and ELISA)	These immunological tests showed to be effective methods for human identification using degraded saliva samples with no enzymatic activity.	[121]/2019
Dental DNA extracts	Dental DNA was extracted via two methods (classic pulverization and decalcification technique and an alternative protocol).	The new procedure could provide a higher amount of good quality DNA from the dental pulp in a short time.	[105]/2019
Dental DNA epigenetic	Methylation and biogeography analyses on Y-Haplotype and mitochondrial DNA (mtDNA) were epigenetic traits used for estimating the age.	DNA methylation and mtDNA analyses could help oral implant investigations for human identification.	[103]/2019
Saliva markers (mtDNA, bacterial DNA, and salivary *α*-amylase)	Saliva samples remaining at a crime scene were treated by three different degradation methods, and their markers' detectability was compared.	Oral Gram+ bacterial DNA analysis and mtDNA typing are represented to be useful for human identification in forensic investigations.	[120]/2019
Saliva-related literature	Systematically summarize the applications of saliva in forensic odontology.	Saliva was confirmed as an efficient and determinative forensic tool.	[122]/2019
Degraded DNA from tooth samples	Three kits were examined for extracting the dental nuclear DNA.	The three-part analysis is shown to be efficient for extracting degraded DNA from tooth samples.	[98]/2020

**Table 3:** Lip print, bite mark, and blood group.

Type	Method	Outcomes	Ref/year
*Lip print*			
Natural dyes (vermilion and indigo) and lysochrome (Sudan black)	The three dyes were applied to visible and latent lip prints and compared using statistical analyses.	All dyes showed comparable results for creating visible and latent lip prints.	[144]/2010
Cheiloscopy	The lip print of a study group comprising 200 subjects was compared.	Lip prints are represented to be individually unique enough for personal identification.	[132]/2011
Lip patterns	A pilot study was performed to evaluate the correlation of lip prints, mandibular canine index (MCI), and fingerprint methods for gender estimation.	These three specific parameters showed no statistically significant correlation.	[123]/2011
Cheiloscopy, palatoscopy, and odontometrics	Lip pattern impression and odontometric measurements were performed for each subject.	The findings confirmed the uniqueness of lip prints that can provide a more reliable sex prediction.	[133]/2014
Lip prints	An experimental model of latent lip prints was provided, photographed, preserved, and analyzed.	The lower lip print revealed a better definition.	[139]/2014
Cheiloscopy using lysochrome	Latent lip impressions were developed using lysochrome.	Lysochrome-printed lip patterns can be preserved in a digital database and are a potential tool for sex determination.	[145]/2015
Lip prints and lip competence	The lip prints were obtained, and the lip competence was determined and recorded.	Lip prints and competence showed specificity for individuals, races, and ethnic groups.	[137]/2015
Cheiloscopy and dactyloscopy	The lip impression, fingerprints, and personal characters of subjects were recorded, and their correlation was statistically analyzed.	The findings showed a fair correlation among the lip/finger traces and gender/personality of subjects suggesting it as an adjunct in forensic investigations.	[124]/2015
Cheiloscopic patterns	Lip prints of subjects were classified based on the Tsuchihashi method, and gender estimation was conducted according to Vahanwala et al.	Cheiloscopy was suggested as a promising supplementary tool for sex determination; however, further standardized studies were suggested.	[131]/2015
Lip prints from different races	Lip prints of 3 ethnic groups were recorded and evaluated.	The 3 races significantly differed in lip patterns.	[140]/2016
Lip outline patterns	Lip outline patterns were impressed on the proforma sheet and analyzed.	Lip outline patterns are represented to be individually unique.	[146]/2016
Morphologic patterns of lip prints	The lip groove patterns of subjects were categorized based on the Tsuchihashi method.	Lip groove patterns showed significant gender dimorphism in the Croatian population.	[10]/2016
Lip and fingerprint patterns	Lip prints and right thumb impressions were recorded and analyzed.	Both these parameters showed to be reliable personal identifiers.	[142]/2016
Lip print	The obtained lip prints were classified into six classes regarding the vertical, horizontal, and intersecting lines.	The outcome revealed no significant correlation between the lip prints and subject identities.	[136]/2017
Cheiloscopy	Lip prints and right thumb impressions were recorded and analyzed.	Both lip and fingerprints were recognized as helpful for sex estimation.	[127]/2017
Oral landmarks	Systematically review the applications of oral landmarks such as bite marks, dental records, and palatal rugae, in forensic identification investigations.	Prosthetic dentistry was discussed to be a significant aid for forensic purposes.	[128]/2017
Lip print patterns	Examine the gender dimorphism in the lip pattern over time using digital photography.	The efficiency of lip prints in forensic investigations for individualization was emphasized.	[126]/2017
Cheiloscopy	The lip print records were categorized and analyzed according to Suzuki and Tsuchihashi methods.	Type I lip pattern was shown as the most frequent type.	[130]/2017
Lip prints and palatal rugae pattern	The records of lip prints and palatal rugae were analyzed based on the Kapali et al.'s classification method.	Lip print (cheiloscopy) is shown to be a more reliable gender identifier than the palatal rugae pattern (rugoscopy).	[141]/2018
Lip print patterns	An algorithm was developed for mass imaginary record processing.	The algorithm facilitated the analysis but lip grooves, and gender showed no association.	[129]/2018
Lip print patterns	Lip print records were collected via a purposive nonrandom sampling method and photographed using a digital camera and analyzed by Photoshop.	Lip prints were shown to be inheritable and various among a population from a single race.	[138]/2019
*Bite mark*			
Overlay generation methods	Overlays were prepared using manual, photocopy, and computer-assisted methods, and the results were compared.	The computer-assisted method was the best one.	[151]/2013
Bite marks on dental casts, bitten objects, and foodstuffs	The bite marks were recorded using x-ray and cone-beam computed tomography (CBCT) and visualized/analyzed by InVivo5® software.	CBCT was proved to be an important aid for forensic applications.	[157]/2013
Bite mark characteristics	A case study was conducted on three crimes with common offenders describing the locations and characteristics of the bite marks.	Bite mark analysis was proved to be an important aid for forensic investigators.	[35]/2014
Topographic overlays	Overlays were generated using the envelopment technique and compared.	The third and fourth cuts were the most reliable sections to be studied.	[160]/2015
Bite mark overlays	Overlays were produced by different methods (e.g., casts, wax impressions, radiopaque wax impression, and xerographic method) and compared.	Every method showed its specificities for bite mark analyses and can be helpful depending on personal preference.	[156]/2016
Bite mark evidence	The article debated the legal requirement of bite mark evidence scientifically discussed its future.	The article reached an unsuccessful outcome for bite mark evidence.	[158]/2016
Bite mark evidence	The opinions of forensic odontologists on bite mark cases were surveyed at the beginning and after 8 weeks.	The results suggested that bite mark evidence is less reliable than other oral landmarks.	[150]/2016
Bite mark models	Positive replicas of bite marks were prepared using computer-assisted modeling methods.	The used computer-assisted method is shown to be simple, reliable, reproducible, and cheap.	[152]/2018
Berry's index (BI)	BI was analyzed in a 300-subject study population.	BI was suggested as a potential aid for bite analysis and facial proportion determination.	[147]/2018
Bite mark evidence	Bite marks were recorded using two materials (styrofoam and wax sheet), analyzed for 3 days, and compared.	Both materials were proper and reproducible, but casts on styrofoam altered after 3 days.	[148]/2018
Bite mark on foodstuffs and inanimate objects	Systematically review the studies analyzing the validity and judicial acceptance of bite marks on foodstuffs and inanimate objects using Daubert rulings.	Because of the vulnerability of forensic procedures, high scrutiny in evaluating such evidence is essential.	[155]/2018
Human bite marks	Assault victims were surveyed using an objective structured questionnaire, and the results were analyzed.	The common occurrence of biting in assault cases should be considered.	[154]/2018
Human bite marks	Three bite mark traits (mesiodistal widths, rotation angles of upper and lower right central incisors, and intercanine distances) were measured and compared with the actual sizes using Photoshop.	Some factors such as skin properties and posture affect the accuracy of measures and interpretation of bite mark injuries.	[149]/2019
*Blood group*			
Extracted dental pulp	The blood grouping and Rhesus (Rh) typing were conducted by slide-agglutination and absorption-elution (AE) technique.	Dental pulp tissue was emphasized as a potential source for blood grouping.	[165]/2012
Soft and hard dental tissues	The reliability of longly stored teeth as a source for blood grouping was assessed by a modified absorption-elution method.	Hard and soft dental tissues could be efficiently used for personal identification.	[166]/2013
Cheiloscopy and blood groups	Any significant association between lip print types and blood groups was searched.	Lip prints and blood groups did not show any correlation.	[125]/2014
Tooth pulp	DNA was extracted from dental pulp tissue of exfoliated primary teeth, and PCR-based blood grouping was done.	PCR is proven to be an effective method for blood grouping.	[162]/2016
Dentin and pulp	The ABO blood grouping and Rh typing were conducted on dentin and pulp of extracted teeth by the AE technique.	The blood grouping showed more sensitivity and significance than the Rh factor typing.	[163]/2016
RBCs and saliva	Secreting status and blood group were simultaneously identified using antibody array, and ABH antigen was detected by surface plasmon resonance (SPR) imaging.	SPR detected almost similar ABH antigen densities both on RBCs and in the saliva.	[70]/2017
Cheiloscopy and ABO blood groups	The association between cheiloscopic patterns/lip print types and blood groups was examined.	The findings showed an association between B+/A+/O- blood groups and type IV lip print and O+/AB+ blood groups and type II lip print.	[161]/2017
Pulpal tissue	The blood group determination was conducted AE method.	The dental pulp is an adequate identifier, especially where teeth are the only practical remnant.	[5]/2017
Dry salivary samples	The results of blood grouping and Rh typing from dry salivary samples were compared with those obtained from the extracted socket.	The results were comparable making dried salivary samples a suitable source for personal identification, especially in mass disasters.	[164]/2018

## Data Availability

This article is a review and does not contain any studies with human or animal performed by any of the authors.

## Abbreviations

3D: Three-dimensional
AAR: Aspartic acid racemization
AE: Absorption-elution technique
AGEs: Advanced glycation end products
AI: Absorption-inhibition
AM: Ante-Mortem
AMEL: Amelogenin protein
AO: Acridine orange
BI: Berry's index
CBCT: Cone Beam Computed Tomography
CEJ: Cementoenamel junction
CH: Coronal height
CPCH: Coronal pulp cavity height
CSI officers: Crime scene investigation officers
CT: Computed tomography
CVM: Cervical vertebral maturation
CVMI: Cervical vertebrae maturation indicators
DI: Demirjian index
DNAm: DNA methylation
DPid: Dental prosthetics identification
DPR: Dental panoramic radiography
DVI: Disaster victim identification
FS: Frontal sinus
Gn-M0: Angle of the intersected lines from the left and right gonion to menton
GUI: Graphical user interface
H/E: Hematoxylin/eosin
ICP: Iterative closest point
IP: incisive papilla
LTR: Long tandem repeats
MCI: Mandibular canine index
MD: Mass disaster
MDCT: Multislice Computed Tomography
MF: Mental foramen
MRI: Magnetic resonance imaging
MS: Maxillary sinus
mtDNA: Mitochondrial DNA
NGS: Next generation sequencing
OF: Oral fluid
OPG: Orthopantomograph
PA: Posteroanterior
PCA: Principal component analysis
PCR: Polymerase chain reaction
PM: Post-Mortem
PV: Pulp volume
RBCs: Red blood cells
RFLP: Restriction fragment length polymorphism
Rh: Rhesus factor
SPR: Surface plasmon resonance imaging
STR: Short tandem repeat
TV: Tooth volume
VNTR: Variable number of tandem repeats
THC: Tetrahydrocannabinol
ASFO: American Society of Forensic Odontology.
